# The bile acid membrane receptor TGR5: a novel pharmacological target in metabolic syndrome

**DOI:** 10.1186/2050-6511-13-S1-A7

**Published:** 2012-09-17

**Authors:** Vanesa Stepanov, Karmen Stankov, Momir Mikov

**Affiliations:** 1 Department of Pharmacology, Toxicology and Clinical Pharmacology, Faculty of Medicine, University of Novi Sad, 21000 Novi Sad, Serbia; 2 Clinical Centre of Vojvodina, Faculty of Medicine, University of Novi Sad, 21000 Novi Sad, Serbia

## Background

TGR5 (M-BAR, GPBAR or GPR131) is a plasma membrane-bound, G protein-coupled receptor for bile acids, expressed in many human cells. The aim of this study was to describe that targeting TGR5 could provide an exciting new pharmacological approach to improve different aspects of the metabolic syndrome in humans.

## Methods

The data on pharmacological targeting of TGR5 have been provided from more than eighty review and original scientific articles, published from 2007 to 2012. The research was performed using the following key words: bile acids, TGR5, metabolism, diabetes, obesity.

## Results

A dietary supplementation of bile acids (BAs) significantly reduced body weight in mice fed with a fat-rich diet. It was the consequence of the induction of deiodinase 2 (D2) through a TGR5/cAMP-mediated pathway. D2 is able to induce the conversion of inactive thyroxine (T4) into the active 3,5,3′-tri-iodothyronine (T3), which enhances the energy expenditure in brown adipose tissue (BAT) and skeletal muscle myoblasts. TGR5 induces glucagon-like peptide-1 (GLP-1) secretion in cultured mouse enteroendocrine STC-1 cells. This property contributes to beneficial effects of TGR5 on glucose metabolism and improves insulin sensitivity. TGR5 activation in mice decreased serum and liver triglyceride levels. The anti-inflammatory action of TGR5 in mouse macrophages attenuated the development of atherosclerotic lesions and could contribute to protective effects of TGR5 on liver steatosis.

## Conclusions

TGR5 may be targeted by natural compounds as well as by synthetic agonists. Despite the fact that targeting TGR5 in animals brings great promise for metabolic syndrome treatment, multiple studies described the side effects of targeting TGR5 and further clinical studies are needed to evaluate and identify safe and efficient TGR5 agonists.

